# Negligible tooth resorptions after anterior open bite treatment using skeletal anchorage with miniplates

**DOI:** 10.1590/2177-6709.25.4.016-022.oin

**Published:** 2020

**Authors:** Genivaldo dos Santos, Alberto Consolaro, Fernanda Meloti, Mauricio de Almeida Cardoso, Ertty Silva, An Tien Li, Monikelly do Carmo Chagas Nascimento

**Affiliations:** 1 Private Practice (Assis/SP, Brazil).; 2 Universidade de São Paulo, Faculdade de Odontologia de Ribeirão Preto, Programa de Pós-Graduação em Odontopediatria (Ribeirão Preto/SP, Brazil).; 3 Universidade de São Paulo, Faculdade de Odontologia de Bauru (Bauru/SP, Brazil).; 4 Faculdade de Medicina e Odontologia São Leopoldo Mandic, Programa de Pós-graduação em Odontologia (Campinas/SP, Brazil).; 5 Universidade de Brasília, Faculdade de Ciências da Saúde, Departamento de Odontologia (Brasília/DF, Brazil).

**Keywords:** Intrusion, Tooth resorption, Anterior open bite

## Abstract

**Introduction:**

When miniplates are used as anchoring for orthodontic mechanics for anterior open bite correction by retraction of anterior teeth and posterior teeth intrusion and retraction, orthodontically induced inflammatory external apical root resorption is clinically negligible.

**Methods:**

A homogeneous sample of 32 patients was used, and the roots of the teeth were compared on CT scans performed before and after orthodontic treatment.

**Results:**

The observed root resorption was minimal, and this can be explained by the uniform distribution of forces in several teeth, simultaneously, in the set of the dental arch and in the bone that supports the teeth.

**Conclusion:**

The most important thing to prevent root resorption in orthodontic practice, besides being concerned with the intensity of the applied forces, is to be careful with its distribution along the roots of each tooth, in the dental arch and in the bone that supports the teeth.

## RESUMO

**Introdução:**

Quando são utilizadas miniplacas como ancoragem para a mecânica ortodôntica de correção da mordida aberta anterior por meio da retração dos dentes anteriores e intrusão e retração dos dentes posteriores, as reabsorções radiculares apicais externas inflamatórias induzidas ortodonticamente são clinicamente irrelevantes.

**Métodos:**

Usou-se uma amostra homogênea de 32 pacientes, e comparou-se as raízes dos dentes em tomografias realizadas antes e depois do tratamento ortodôntico.

**Resultados:**

As reabsorções radiculares observadas foram mínimas, e isso pode ser explicado pela distribuição uniforme das forças em vários dentes, simultaneamente, no conjunto da arcada dentária e no osso que suporta os dentes.

**Conclusão:**

O mais importante para se prevenir as reabsorções radiculares na prática ortodôntica, além de se preocupar com a intensidade das forças aplicadas, é tomar cuidado com a sua distribuição ao longo das raízes de cada dente, na arcada dentária e no osso que suporta os dentes.

Tooth resorption, one of the factors that limit tooth movements, should be predicted and minimized during orthodontic treatment, and its predicting factors should be identified. Among these factors are the morphology of root, apex and bone, as well as the use of intrusive mechanics and intermaxillary elastics, for example.^1^ ^,^ ^2^

Intrusion without an ideal anchorage may become one of the most complex and resorptive procedures, because of undesirable collateral movements^3^ ^-^ ^5^. Intrusion, uncontrolled pendular inclination, and translation of cortical bone are the most resorptive procedures,^6^ ^,^ ^7^ particularly when torque is necessary to control them.^8^ ^,^ ^9^ In fact, the forces applied during the use of intrusive mechanics, a predicting factor of tooth resorptions during orthodontic treatment, lead to inclination, and not actual intrusion. An intrusive force is perpendicular to the long axis of the tooth and forms a 90-degree angle to the bottom of the alveolus, which does not occur in clinical practice. Tooth intrusion into bone is obtained by a tipping movement, because of the inclination of anterior teeth and the root bifurcation and trifurcation angles.^5^

Force increases do not mean that the velocity of tooth movement also increases, and such forces usually lead to an increase in inflammatory external apical root resorption induced by orthodontic treatment.^10^ The distribution of low intensity forces, if restricted to a single focal area, leads to the death of cementoblasts.^6^ Uniform force distribution along the roots is more important than force intensity in determining the frequency and severity of external apical resorptions.^6^

The use of mini-implants as temporary anchorage devices (TAD) for intrusion has some advantages, such as their easy placement and removal in different areas, as well as their low cost. However, they may affect orthodontic movements when placed in the alveolar process between tooth roots. Moreover, they may not withstand forces greater than 150-350 g, depending on the type of bone and mini-implant diameter.^4^ ^,^ ^11^ Even when placed in the infrazygomatic crest or above the external oblique ridge of the mandible, that is, on the buccal shelf, these forces are limited when compared with the ones that miniplates may withstand. 

Miniplates have been developed as alternatives for larger anchorage needs. Placed in the basal bone, miniplates do not affect tooth movement, but have a greater stability and withstand much greater forces, which may be simultaneously applied on the three spatial planes - transverse, vertical and horizontal.^3^ ^,^ ^7^ ^,^ ^13^ In some cases, they may be used as an alternative to orthognathic surgery, as they lead to successful bone remodeling of the dental arches.^6^ ^,^ ^7^ ^,^ ^13^ ^,^ ^14^ ^,^ ^15^

Intrusion of posterior tooth using miniplates as anchorage may be a good alternative, because tooth movement during orthodontic treatment is greater than when conventional techniques are used^10^. The use of miniplates simplifies the complexity of intrusion and prevents undesired lateral movements. At the same time, it reduces the frequency of inflammatory external apical root resorption induced by orthodontic treatments. 

External apical resorptions in cases treated without miniplates may be frequent and severe. Therefore, the present study used cone-beam computed tomography (CBCT) scans of patients with an anterior open bite treated with posterior tooth intrusion to evaluate external apical resorptions in treatments with skeletal anchorage with miniplates placed in each posterior quadrant of the dental arches. 

This study describes and analyzes the results of a investigative clinical study that found that the use of miniplates for orthodontic movements induces negligible external inflammatory apical resorptions^16^.

## METHODS

CBCT scans obtained before and after orthodontic treatment for 32 patients (23 women) with anterior open bite were selected. Minimum age was 16 years, and maximum, 55 years. Measurements before and after orthodontic treatment were made twice by a single calibrated observer at a 30-day interval. After their orthodontic treatment, the patients had a molar and canine Angle Class I relationship, and their open bite had been closed. 

**Figure 1:**
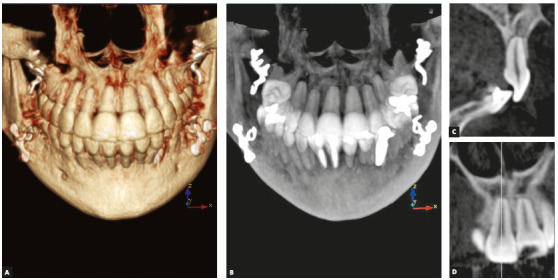
Images of the patient with the largest orthodontically induced inflammatory external apical resorption in the sample studied, after the treatment of the anterior open bite with miniplates.

The roots of all teeth in the maxilla and mandible were measured on oblique sagittal and coronal slices, using the long axis of the root as a reference, from the apex to the cervical line, at the cementoenamel junction, in the buccolingual direction. The CT images obtained using an iCAT Classic scanner (Imaging Science, Hatfield, PA) were retrieved from a database for a 3D orthodontic diagnosis and miniplate placement and removal (Fig 1). 

The “T” miniplates were placed in the region of the left and right infrazygomatic crest in the maxilla and in the posterior region of the external cortical bone of the mandible, at the external oblique line. The patients were treated using the same protocol:^15^ standard Ricketts prescription brackets with 0.018 x 0.028-in slots (Forestadent, Pforzheim, Germany) and four miniplates placed in the left and right maxilla and the left and right mandible. Leveling, aligning and moving teeth distally were performed using a progressive increase in wire caliber: 0.012-in nickel-titanium (NiTi) (Forestadent, Pforzheim, Germany), 0.016 x 0.016-in 80-g Neo Sentalloy (Dentsply Sirona, São Paulo, Brazil), 0.016 x 0.016-in 80-g Titanol low force (Forestadent, Pforzheim, Germany), 0.016 x 0.022-in 120-g Titanol low force (Forestadent, Pforzheim, Germany), 0.016 x 0.016-in Blue Elgiloy (Rocky Mountain Orthodontics, Denver, CO) and 0.016 x 0.022-in Blue Elgiloy (Rocky Mountain Orthodontics, Denver, CO). 

All posterior maxillary and mandibular teeth were moved distally using activation every three weeks. Distalization began with a 0.012-in NiTi wire, Ultra Thread Round Solid (GAC) elastomeric ligatures with a diameter of 0.030-in, tied from the miniplates to the teeth or wire, depending on the force vector necessary; force was 150-200 g for each elastomeric ligature. Subsequently, after heat-activated 0.016 x 0.016-in wires had already been inserted, the sliding-jigs were adjusted. The size of e-links (TP Orthodontics, Campinas, Brazil) was the same as the distance from the miniplate to the sliding-jig, which generated a force ranging from 100 g to 400 g for molar distalization. At this phase, according to the need of posterior intrusion and using a 150 to 200-g force, elastomeric ligatures were extended from the miniplate to the posterior part of the sliding-jig and placed in the mesial area of the first molar, or tied directly to the molar tube. Class II elastics may be used, if necessary, from the maxillary canines to the mandibular miniplates to generate a force of about 50-100 g, depending on the discrepancy of the anterior open bite. 

The anterior teeth were retracted using a segmented 0.016 x 0.016-in Blue Elgiloy wire (Rocky Mountain Orthodontics, Denver, CO) with a "C"-shaped hook at each end and placed in the disto-cervical region of the canines. A wire segment was inserted in the same slots in the anterior teeth, which already had the 0.016 x 0.016-in 80-g Titanol low force wire (Forestadent, Pforzheim, Germany). Immediately after that, elastomeric ligatures were selected, inserted in the miniplates and extended to the "C" hook of the wire segment, to retract the anterior teeth and close the space between the canines and premolars using a force of 150-300 g on each side. The cases that required space closure between the lateral incisors and canines received a modified Ricketts prescription retraction archwire fabricated using a 0.016 x 0.016-in Blue Elgiloy wire (Rocky Mountain Orthodontics, Denver, CO) and activated at its ends using forces ranging from 60 to 100 g. 

The DICOM files for each patient were imported to the DTX Studio Implant 3.3.3.1 software (Nobel Biocare, Zurich, Switzerland). A more accurate analysis was conducted using image manipulation tools for brightness, contrast and filter adjustment. First, each tooth was positioned according to its long axis on oblique sagittal and coronal slices. These slices included the most central area of the tooth, so that the root apex and the crown were visualized. In the cervical area, two dots were placed at the cementoenamel junction: one on the buccal surface and the other on the lingual surface. The buccolingual line formed from one dot to the other was called cervical line. The intersection of the cervical line on the oblique coronal and sagittal slices formed a point in the cervical area called “cervical point”, automatically determined by the software and visualized on the oblique coronal slice. 

The values obtained were analyzed statistically using the SPSS for Windows 24.0 (IBM Corp., Armonk, NY). The Shapiro-Wilk test for normality was used to determine data distribution. As data distribution was normal, a paired *t*-test was used to compare root length before and after treatment. Measurements for the calculation of method error were made twice for the phases before and after treatment, at an interval of 30 days between measurements. Analyses were conducted using the mean value for the first and second measurements to reduce procedural errors. The formula developed by Dahlberg was used to estimate random error.

## RESULTS

The difference in root length in the groups of anterior and posterior teeth before and after orthodontic treatment was statistically significant (*p*< 0.01), demonstrating a mean 0.85-mm resorption for anterior teeth and 0.69-mm for posterior teeth. Posterior root resorptions were a mean 1 mm smaller for all teeth after intrusion anchored to miniplates. All posterior teeth had resorptions of less than 1 mm. In the group of anterior teeth, 50% had resorptions smaller than 1 mm, and the rest, slightly greater than 1 mm, at a maximum of 1.17 mm. 

The analysis of intraobserver agreement in the evaluation of anterior teeth revealed a statistically significant difference for teeth #32 (*p*= 0.018, 0.19 mm) and #43 (*p*= 0.018, 0.19 mm). In the posterior teeth, a significant difference was found for premolars #34 (*p*= 0.002 - 0.29 mm) and #35 (*p*= 0.009, 0.22 mm), as well as for molars #17 (*p*= 0.037, 0.14 mm) and #26 (*p*= 0.042 - 0.32 mm). There was a systematic error, that is, *p*-value was < 0.05, in the measurement of these teeth. However, the measurement error was up to 0.32 mm only, which is not clinically significant. 

## DISCUSSION

Although intrusion was performed in the posterior region, the anterior teeth had a greater level of inflammatory external apical root resorption induced by orthodontic treatment. This may be explained by the need to retract the anterior teeth using sliding mechanics and torque control, which was achieved using the auxiliary archwire segment and forces ranging from 150 g to 200 g. 

Some cases also required the use of a modified Ricketts prescription retraction archwire (60-100 g) and Class II elastomeric ligatures (50-100 g). Intrusion, pendular inflection and translation of the cortical bone are the most resorptive movements.^6^ ^,^ ^7^ The factors that contributed to inflammatory external apical root resorption induced by orthodontic treatments in the anterior teeth were:

a) The forces were applied to the crowns, far from the tooth center of resistance, an inherent factor of all orthodontic techniques.
b) The natural inclination of teeth.^9^ ^,^ ^14^ ^,^ ^17^
c) The anatomic root characteristics, which predict inflammatory external apical root resorption induced by orthodontic treatment in the groups of incisors.
d) The concentration of forces in the apical third, because of the absence of bone deflection.
e) The greater movement of incisors during orthodontic treatment.^8^ ^,^ ^9^

Intrusion performed according to conventional Orthodontics has results of little clinical significance when compared with other types of movement. Its limitations produce undesired movements, which contribute to an increase in treatment time and more severe resorptions.^7^ ^,^ ^9^ Therefore, it is classified as the most complex mechanics, and its resorptive potential is accentuated with the increase of force application time and the inclusion of torque to control it.^18^

In this study, posterior teeth intrusion used forces of 150-200 g applied and distributed to the groups of posterior teeth and reactivated every 21 days. In proportion, the ideal force for the intrusion of posterior teeth corresponds to the force inside a blood capillary vessel, which includes light and heavy forces of 25 g and 225 g. Treatment time, intermittent or continuous force application and force intensity affect the level of inflammatory external apical root resorption induced by orthodontic treatment. *In vitro* studies showed a low level of this type of inflammatory resorption when the treatment includes intrusion, mini-implants as TAD and forces of 50-200 g.^5^ ^,^ ^10^ Intrusion results were significant, and inflammatory external apical root resorption induced by orthodontic treatments was not always found. 

Quantitative studies evaluated the level of root resorption using mini-implants as TAD, and examined all maxillary and mandibular roots before and after the intrusive treatment with forces ranging from 200 g to 300 g and reactivated every 15 days. They found that the presence of inflammatory external apical root resorption induced by orthodontic treatment was statistically significant, but, because resorptions measured 0.34 mm to 0.74 mm only, they were not clinically significant. 

A study with dogs^3^ to investigate posterior tooth intrusion using miniplates and forces of 100-150 g found inflammatory external apical root resorption induced by orthodontic treatments measuring 0.1 mm into cementum four months after the beginning of the treatment, and the results of intrusion were significant. In a study with patients, mean differences of 0.5 mm between root length before and after treatment were found, but these results were not clinically significant.^22^

Although mini-implants and miniplates produce similar external inflammatory resorption and intrusion, mini-implants as TAD have limitations. They affect tooth movements when placed in the alveolar bone, between tooth roots and, mainly, they do not withstand very high forces, of 150-350 g,^11^ not even when placed in the infrazygomatic crest or above the external oblique line of the mandible, on the buccal shelf.^12^ ^,^ ^14^

Miniplates are recommended for more complex cases that require more extensive movement. As they withstand greater forces, simultaneous tooth movements in the transverse, vertical and horizontal planes can be attempted, and clinical results are better than those obtained when using mini-implants as TAD.^3^ ^,^ ^6^ ^,^ ^7^

Miniplates also affect all the extent of the maxilla and mandible, and the side effects of bone remodeling produced by miniplates contribute to the correction of anterior open bites, reducing treatment time^15^. Lateral radiographs of treatments using skeletal anchorage with miniplates for intrusion revealed a significant 1.76-mm intrusion of maxillary molars and non-significant inclination, with a reduction of the anterior facial height, counterclockwise rotation of the mandible and changes in the occlusal plane.^23^ In extremely complex cases, the indication of miniplates may be a valid non-surgical treatment alternative. The use of miniplates results in bone remodeling in cases for which not even orthognathic surgery would be an ideal solution.^5^ ^,^ ^14^

Few studies have investigated the magnitude of inflammatory external apical root resorption induced by orthodontic treatments associated with posterior tooth intrusion using skeletal anchorage with miniplates. This study is, to our knowledge, the first to clinically evaluate all posterior teeth in treated individuals. The amount of root resorption in all teeth was analyzed using 0.4-mm voxel CBCT scans, including images previously obtained. These images were used to make a diagnosis and a 3D orthodontic treatment plan. Although this voxel size is not classified as high resolution, studies about root resorption found no statistic differences when smaller voxels were used, particularly when resorption is in the apical third of the root.^25^ ^,^ ^26^

Further CT studies should measure posterior tooth intrusion using skeletal anchorage with miniplates and after bone remodeling, to evaluate the effects of this technique for the correction of anterior open bite. 

## FINAL CONSIDERATIONS

External orthodontically-induced inflammatory apical root resorptions were clinically negligible after orthodontic treatment to correct anterior open bite by retraction of anterior teeth and intrusion and retraction of posterior teeth anchored in miniplates. 

The distribution of uniform forces to several teeth simultaneously may explain why the apical resorptions associated with orthodontic movement were negligible when using miniplates for skeletal anchorage. This technique reduces the chances of vascular compression in the periodontal ligaments, which would lead to the death of cementoblasts, exposure of the mineralized portion of the root and attraction of clasts, and therefore, with consequent root resorptions. These findings suggest that the most important step to prevent root resorptions in orthodontic practice is to pay attention not only to the intensity of forces applied, but also, and more importantly, to their distribution to the roots of each tooth, the dental arch and the bone that supports the teeth.
